# Patellofemoral Cartilage Repair

**DOI:** 10.1007/s12178-018-9474-3

**Published:** 2018-05-18

**Authors:** Alexandre Barbieri Mestriner, Jakob Ackermann, Andreas H. Gomoll

**Affiliations:** 1 Cartilage Repair Center, Department of Orthopaedic Surgery, Harvard Medical School, Brigham and Women’s Hospital, 850 Boylston Street, Suite 120, Chestnut Hill, MA 02467 USA; 2 Federal University of São Paulo, Paulista School of Medicine, São Paulo, Brazil

**Keywords:** Patellofemoral joint, Chondral lesion, Cartilage repair, Osteochondral allograft, Autologous chondrocyte implantation, Particulated allograft

## Abstract

**Purpose of Review:**

This review provides an overview of well-established and newly developed cartilage repair techniques for cartilage defects in the patellofemoral joint (PFJ). An algorithm will be presented for approaching cartilage defects considering the distinct anatomy of both the patellar and trochlear articular surfaces.

**Recent Findings:**

Recent studies on cartilage repair in the PFJ have demonstrated improved outcomes in an attempt to delay or obviate the need for arthroplasty, and improve symptoms in young patients. While autologous chondrocyte implantation shows good and excellent outcomes for chondral lesions, osteochondral defects are adequately addressed with osteochondral allograft transplantation. In case of patellar malalignment, concomitant tibial tubercle osteotomy can significantly improve outcomes. Particulated cartilage and bone marrow aspirate concentrate are potential new alternative treatments for cartilage repair, currently in early clinical studies.

**Summary:**

Due to the frequency of concomitant anatomic abnormalities in the PFJ, a thorough clinical examination combined with careful indication for each procedure in each individual patient combined with meticulous surgical technique is central to achieve satisfying outcomes. Additional comparative studies of cartilage repair procedures, as well as investigation of newer techniques, are needed.

## Introduction

The management of articular cartilage defects remains a challenging clinical problem for orthopedic surgeons. Articular cartilage is a highly organized tissue with complex biomechanical properties and substantial durability. However, it has poor intrinsic capacity for healing, and defects can lead to considerable functional impairment, subsequent joint degeneration, and development of osteoarthritis (OA) [1]. In the United States (US) alone, OA is a condition that affects up to 27 million people with an estimated economic cost of 89 billion US dollars annually [2].

The patellofemoral joint (PFJ) is frequently involved in symptomatic cartilage disease of the knee [3]. A recent meta-analysis revealed that, based on magnetic resonance imaging (MRI), up to 52% of patients with knee pain or symptomatic OA of the knee are diagnosed with cartilage lesions in the PFJ [4•]. Moreover, patellofemoral OA has been associated with symptoms of pain, stiffness, and functional limitations [5]. The pathomechanism of patellofemoral OA is multifactorial, including chronic repetitive microtrauma due to suboptimal extensor mechanism alignment, and acute macrotrauma, most commonly patellar dislocations, which are associated with cartilage defects in up to 95% of patients [6, 7••].

Chondral lesions on the patellar and trochlear articular surfaces are particularly challenging to manage due to the complex biomechanical environment and substantial load transmission through the PFJ during weight-bearing activity [8].

First-line conservative treatment of cartilage lesions, in general, aims to relieve inflammation and controlling pain to regain functional capacity. This can be achieved with nonsteroidal anti-inflammatory medications, intra-articular corticosteroid injections, and hyaluronic acid viscosupplementation [8]. In addition to muscle strengthening to improve absorption of physiological loads across the knee, weight loss and activity modification to avoid aggravating pain and functional limitation may improve symptoms [9, 10]. Conservative treatment of patients with anterior knee pain should be attempted for at least 3 months before considering surgical intervention, as most patients will experience pain relief with physical therapy and can therefore avoid surgical treatment [11, 12].

Surgical treatment options for focal cartilage lesions in the knee include debridement/chondroplasty, bone marrow stimulation, autologous chondrocyte implantation (ACI), osteochondral autograft transfer (OAT), osteochondral allograft transplantation (OCA), and newer techniques such as particulated cartilage procedures (autograft and juvenile allograft). Choosing a surgical treatment is based on morphological factors of the chondral lesion such as the location within the knee joint (femoral condyles vs. patellofemoral), location on the involved articular surface (i.e., inferior vs. superior patellar pole), size, depth (involvement of the subchondral bone), containment (contained vs. not contained), as well as on patient characteristics like age, body mass index (BMI), limb and extensor mechanism alignment, and activity level.

Also, adequate treatment of concomitant pathologies is crucial to the success of any cartilage repair in the knee. This review will concentrate solely on cartilage lesions in the PFJ, and therefore, tibial tubercle osteotomy (TTO) will be discussed briefly.

First described for the treatment of patellar instability [13], anteromedialization of the tibial tubercle has also been found to unload the PFJ [14]. The procedure can improve patellofemoral contact mechanics in patients with patellofemoral malalignment by transferring contact forces from distal to proximal and from lateral to medial on the patellar articular surface [15•]. Hence, this procedure is especially valuable in patients with chondral lesions on the inferior pole or lateral facet of the patella [12]. Due to its significant positive impact on clinical outcomes, TTO is frequently performed concomitantly with patellofemoral cartilage repair in patients with PF maltracking [7••, 16–19, 20••].

This article provides a review of the current state of cartilage repair in the PFJ, focusing on well-established, as well as emerging techniques meeting the distinct requirements of cartilage repair in the PFJ. Moreover, we will provide an algorithm to adequately address patellofemoral cartilage defects.

### Chondroplasty

Chondroplasty, also referred to as cartilage debridement, is one of the most performed procedures involving cartilage [24]. The goal of chondroplasty is to transform an irregular and unstable cartilage lesion into a more regular and stable construct. It is typically indicated for partial or full-thickness chondral lesions smaller than 1–2 cm^2^ but can also be performed in larger lesions preparing the site for a subsequent cartilage repair procedure such as ACI [25]. In the senior author’s practice, most patients undergo chondroplasty during the cartilage biopsy in the first stage of an elective ACI for the PFJ and/or tibiofemoral joint (TF).

It appears of importance to create a regular lesion with stable vertical walls. However, the negative effects on cartilage viability beyond the area of resection remain controversial, and therefore, overly aggressive debridement should be avoided [26–30]. Surgeon’s attention is critical to neither transform a contained into an uncontained lesion nor to needlessly expose the subchondral bone [31].

Reports of clinical outcomes for chondroplasty in the PFJ remain limited. Anderson et al. [32•] retrospectively studied 86 patients who were submitted to isolated chondroplasty for chondral lesions in the knee with a mean size of 3.3 cm^2^ and ICRS grades 2–4. The majority of these patients had lesions in the PFJ (58.5%). The authors found that patients with lower preoperative scores gained more benefit from chondroplasty, but no correlation between anatomic location and amount of improvement could be observed. Federico and Ryder [33] reviewed the records of 36 patients who underwent arthroscopic debridement for chondral lesions of the patella. While 57.9% of patients with traumatic chondromalacia had good or excellent results, only 41.1% with atraumatic chondromalacia showed good or excellent results after chondroplasty.

### Bone Marrow Stimulation

Bone marrow stimulation refers to any technique that promotes the migration of pluripotent mesenchymal stem cells from the subchondral bone to the chondral defect surface, thereby promoting fibrocartilage repair. The most popular techniques are the Pridie drilling technique, using K wires, and the microfracture technique described and popularized by Steadman and colleagues, which uses angulated awls [34, 35].

While full thickness and contained chondral lesions of up to 4 cm^2^ are an acceptable indication for performing microfracture in the TF, a lower threshold (2 cm^2^) should be considered for the PFJ. This is due to the distinctive biomechanics and higher sheer stresses in this joint, which would affect durability of the formed fibrocartilage. Furthermore, superior outcomes were observed in patients younger than 40 years, BMI under 30 kg/m^2^, and duration of symptoms less than 12 months. Importantly, microfracture should be avoided in uncontained chondral lesions [36].

**Fig. 1:**
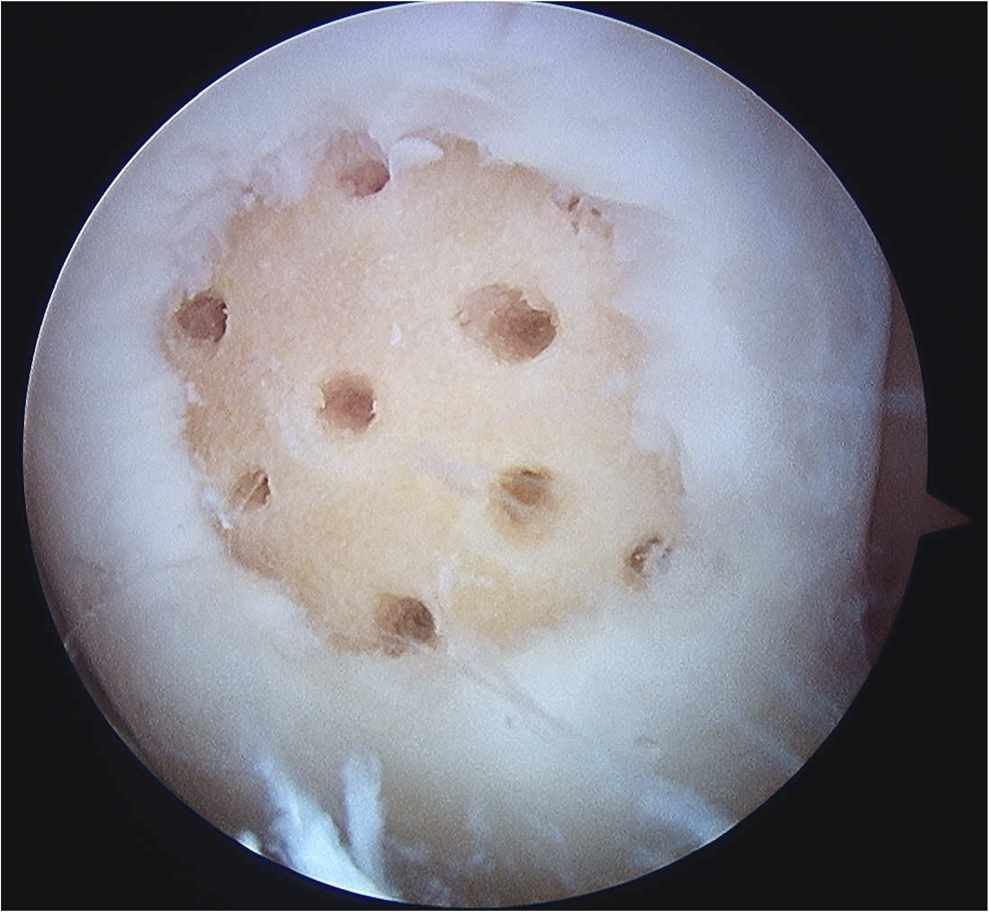
Arthroscopic image of a small, well-contained chondral lesion treated with microfracturing. Note that the holes are 3–4 mm apart from each other to prevent tunnel collapse

Surgical principles remain the same regardless of the chosen technique and are paramount for success. It is essential to perform a thorough debridement all the way down to the subchondral bone, including the layer of calcified cartilage, with stable, verticals walls to create adequate containment. Next, the perforations must be perpendicular to the bony surface, at least 3 mm deep, and spaced 3 to 4 mm apart from each other (Fig. 1). This will cause bleeding, clot formation, and the migration of the pluripotent mesenchymal stem cells into the cartilage defect. As a result, fibrocartilage with type I collagen will be produced that has, however, inferior biomechanical properties compared to the type II collagen typical for native hyaline cartilage. While lesions in the trochlea can be easily addressed by arthroscopy, patellar defects are more challenging to approach. A small arthrotomy may be necessary for lesion visualization and proper instrument angulation. Additionally, counter pressure on the anterior aspect of the patella may be needed during bone marrow stimulation due to greater mobility and harder bone of the patella.

Kreuz et al. [37] evaluated 85 patients who underwent microfracture and compared the outcomes of different anatomic locations within the knee. The authors concluded that microfracture results in short-term improvement followed by a sharp decline in outcomes after 2 years of follow-up. Interestingly, the functional outcomes in patients with patellofemoral chondral lesions were worse regardless of follow-up. Mithoefer et al. [36] showed that the failure rate was up to 6% in the first 2 years, with an increase of up to 31% after 5 years of follow-up. Minas et al. [38] demonstrated a 3-fold increase in failure rate of ACI after a previous marrow stimulant procedure compared to primary ACI (26 vs. 8%), showing that microfracture can negatively affect the outcomes of subsequent procedures. Consequently, the senior author performs a bone marrow procedure only in small chondral lesions (< 2 cm^2^) of the PFJ (and mostly limited to the trochlea), thus maximizing the likelihood of a successful outcome without the need for subsequent procedures.

**Fig. 2:**
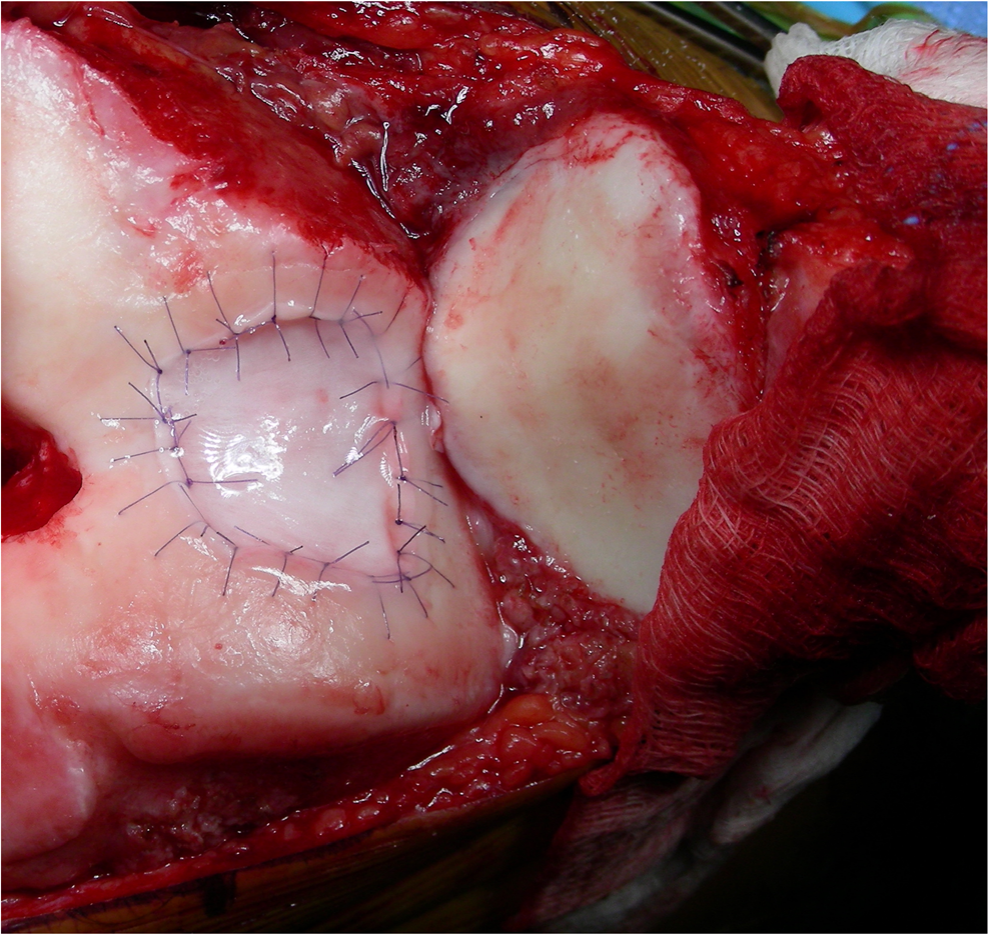
Intraoperative image of an autologous chondrocyte implantation (ACI) to the central trochlear. The collagen membrane is secured with 6-0 absorbable sutures and watertight sealed with fibrin glue

Recently, bone marrow aspirate concentrate (BMAC) has emerged as an alternative treatment option. Though its application is still under investigation, BMAC has the advantage of not damaging the local subchondral bone while allowing higher numbers of mesenchymal stem cells to be brought into the defect. The bone marrow clot is implanted into the defect under a membrane cover to stabilize the reparative tissue. [46•]. BMAC can also serve as a biological augmentation for other procedures such as OAT and OCA (Fig. 2). Hopefully, our understanding of the clinical benefits of BMAC will increase with future results of ongoing studies [46•, 47].

### Autologous Chondrocyte Implantation

ACI is one of the senior author’s most performed procedures for chondral lesions in the PFJ. It allows the treatment of larger defects and matching of the complex contour of the patellar and trochlear articular surfaces. With more than 25 years of experience, ACI technology has evolved but the two-stage technique principles remain the same [48]. The first stage involves the arthroscopic harvesting of 100–300 mg of full-thickness articular cartilage from non-weight-bearing areas of the femoral condyle, or the superior and lateral margin of the intercondylar notch. In vitro, the cartilage matrix is processed and the chondrocytes are cultured for at least 2 weeks. During the second stage, the chondral defect is debrided and the cultured chondrocytes are implanted. The debridement must be performed carefully, avoiding damage to the subchondral plate and creating stable vertical walls. Then, the chondrocyte-seeded collagen membrane is placed in the defect and secured with either fibrin glue alone in case of excellent containment or fixed to the surrounding cartilage with 6-0 absorbable sutures. (Fig. 2).

The original technique, also referred to as first-generation ACI, utilized a periosteal patch that was harvested from the patient’s proximal tibia [49]. As a result of overgrowth issues and inferior outcomes, periosteal patches were progressively replaced by collagen membranes—bilayered synthetic biodegradable scaffolds for ingrowth of chondrocytes—which formed the second-generation ACI [50, 51]. Aside from the containment function, scaffolds have the potential of in vivo chondroinduction and chondroconduction [52]. In the current third-generation ACI, also known as matrix-induced autologous chondrocyte implantation (MACI), the chondrocytes are, again, cultured in vitro but replicated inside a three-dimensional scaffold membrane [53••]. While comprising the same scaffold associated potentials, the application of MACI also simplifies the surgery by omitting both the chondrocyte injection underneath a membrane and the watertight sealing to ensure chondrocyte containment within the lesion. While studies have demonstrated reduced rates of re-operation in membrane ACI compared with periosteal ACI, further comparative studies are needed to show superiority among these techniques [54, 55•, 56].

ACI is indicated in full-thickness chondral lesions larger than 3–4 cm^2^ and can be performed in the TF and PFJ. Preoperative assessment of the subchondral bone on MR-imaging is of critical importance. Patients with subchondral plate alterations may require concomitant bone grafting (“sandwich” technique), or osteochondral transplantation in order to address the subchondral bone. ACI should be avoided in patients with previous bone marrow stimulation procedures and significant subchondral changes such as cystic degeneration and extensive subchondral edema [38, 50].

As in other cartilage repair techniques, it is important to achieve good containment of the chondral defect prior to ACI. In patellar lesions, however, this can be more challenging to accomplish due to the unique anatomy of its articular surface. To resolve this issue, ACI can be performed in conjunction with OAT to ensure proper containment [57]. Contraindications for ACI include inflammatory arthritis, obesity, and smoking. Although considered a contraindication for primary treatment, bipolar lesions can be addressed with ACI as a salvage procedure exhibiting encouraging results [58••, 59••, 60].

In contrast to other cartilage procedures, ACI for the PFJ has been extensively studied in the literature. Initial reports of the outcome after ACI in the PFJ were disappointing [49]. Improved ACI techniques and the treatment of concomitant patellofemoral malalignment, along with better understanding of the patellofemoral biomechanics, led to enhanced outcomes in recent years [61•, 62–64, 65•, 66]. Gomoll et al. [6] evaluated 110 patients with patellofemoral ACI in a multi-center study across the US. A total of 84% of patients had good or excellent results after a minimum of 4-year follow-up. Similar results were obtained by von Keudell et al. [21••], showing that 83% of patients had good or excellent functional outcomes after ACI to isolated chondral lesions of the patella with a failure rate of 10% after 15 years of follow-up. As previously mentioned, lateral facet or inferior pole patellar lesions should be addressed with ACI in combination with an anteromedializing TTO to achieve improved outcomes [17, 19, 67•].

ACI sandwich technique refers to the use of two membranes with impaction grafting of the subchondral bone deficit. It has shown excellent results in the treatment of osteochondral defects on the femoral condyles [68, 69], but further studies must investigate its eligibility as primary option in the PFJ.

Lately, arthroscopic techniques for ACI have emerged showing encouraging results. Ebert et al. [70•] reported 90% of good or excellent outcomes after arthroscopic MACI with 5 years of follow-up. In contrast, Biant et al. [71•] demonstrated a 16-fold increase in cellular viability when ACI was performed using a mini-arthrotomy compared with an arthroscopic approach, suggesting that the arthroscopic procedure has the potential to negatively affect chondrocyte viability.

In a recent systematic review, Andriolo et al. [22••] evaluated 58 studies and found an overall failure rate of 14.9% for ACI across the knee joint, most of them occurring in the first 5 years without differences between ACI and MACI techniques. The authors criticized the heterogeneous description of failure among all studies and underlined the importance of a coherent definition of failure. Another systematic review looked at MACI outcomes in a 5-year follow-up period and found a significantly higher failure rate in the TF compared to the PFJ (12.4 vs. 4.7%) [23•] (Table 1)

**Table 1:** Results of ACI for the treatment of chondral lesions in the PF

Study	LE	Cartilage repair technique	No. of patients	Mean patient age in years (range)	Mean follow-up (range)	Defect size, mean in cm^2^	Outcomes
Gomoll et al. [7••]	IV	ACI	110	33 (15–55)	90 months (48–192)	5.4	84% good or excellent results, 9 failures (8%) which were treated with partial or total arthroplasty
von Keudell et al. [21••]	IV	ACI	30	32 (15–49)	7.3 years (2–15)	4.7	Good to excellent results in 25 (83%) patients, fair in 4 (13%) patients, poor in 1 (3%) patient, and 3 failures which were treated with PF and bicompartmental arthroplasty
Ebert et al. [20••]	III	MACI	194	37.7 (TF) vs. 37.9 (PF)	2 years	3.1 (TF) vs. 3.0 (PF)	MACI in PF and TF shows similar results when PF maltracking is corrected if needed
Andriolo et al. [22••]	IV	ACI	4294	29 (21–51)	5–12.3 years	4.4	Failure rate of 14.9% (0–42.5) at 86 months. 64.0% of failures happened in the first 12 months, 26.1% between the second and fifth year, and 9.9% after the fifth year
Schuette et al. [23•]	IV	MACI	578	31.3–42.3	Minimum 5–10 years	2.3–5.3	All scores significantly improved compared to baseline. In total, 9.7% of all patients failed, including 4.7% of PF patients and 12.4% of TF patients

.

ACI remains a costly treatment option for patients with chondral defects in the knee. Though, the procedure cost-effectiveness seems to be inside the range of other cartilage procedures, considering the substantial delay of other more aggressive and expensive procedures such as partial of total knee arthroplasty [72, 73•, 74•].

### Osteochondral Autograft Transfer (OAT)

**Fig. 3:**
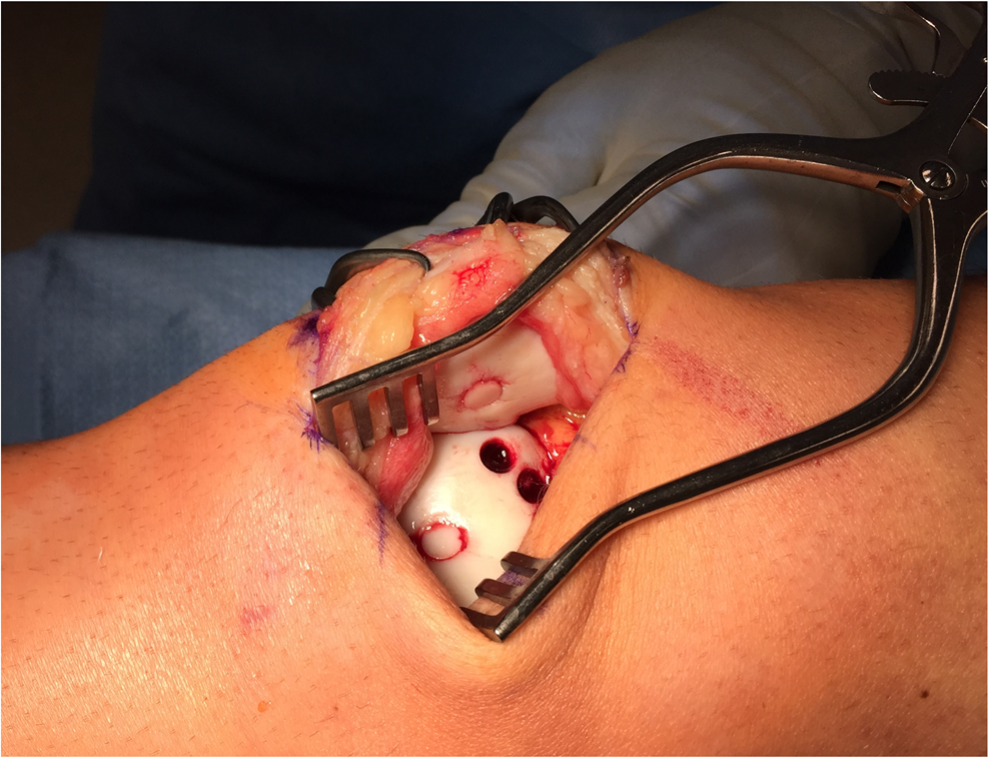
Intraoperative image of two osteochondral autograft plugs transferred to both the patella and trochlea. Note that the lateral margin of the trochlea served as donor site to harvest both osteochondral cylinders

This technique describes the harvesting of 10–15 mm deep osteochondral cylinders from healthy, non-weight-bearing areas of the knee, typically the medial or lateral margins of the trochlea, posterior femoral condyles, or intercondylar notch, and transferring them to the defect site of the ipsilateral knee (Fig. 3). While OAT has the advantage of transferring hyaline cartilage in a one-stage procedure with good bony integration and no risk of immunologic complications, donor site morbidity remains a concern in OAT procedures. This limits the indication for OAT to only small cartilage lesions up to 2–3 cm^2^ in size [75]. In a biomechanical study, Garretson et al. [76] found that the medial trochlea and distal lateral trochlea had the lowest contact loads and hence could provide desirable cylinder grafts for the PFJ.

Precise graft fitting and creating of a smooth articular surface in the PFJ are essential to achieving satisfactory results in patients after OAT. In fact, graft prominence of more than 1 mm has shown to be associated with less favorable outcomes, and patients frequently describe persistent catching sensations months after the surgery [77, 78, 79•].

This is particularly challenging in the PFJ as a result of two issues: The unique anatomy of the patellar and trochlear surfaces complicates forming a smooth articular surface; every donor graft will have a thinner cartilage layer than the surrounding patella and thus, results in local cartilage bone interface mismatch [80].

Some authors reported up to 92% good to excellent results in patients with chondral lesions on the femoral condyle, while lesions in the PFJ showed 79% good to excellent results [81]. Especially patellar lesions have been reported to be associated with less favorable results with up to 100% failure rate after 1 year follow-up, which is thought to be due to the previously described anatomic characteristics of the PFJ [39•, 82]. Yet, other authors believe that patellofemoral malalignment plays an important role in the outcome of OAT to the patella. They found significant improvement in clinical outcomes and 100% graft integration after 12 months follow-up in patients with small (< 2.5 cm^2^) full-thickness chondral lesion of the patella without malalignment of the PFJ [40••]. Recently, Yabumoto et al. [79•] stated that if high congruity is achieved through meticulous implantation of donor grafts perpendicular to the articular surface, favorable results can be obtained for chondral lesions in the PFJ.

### Osteochondral Allograft Transplantation

Osteochondral Allograft Transplantation (OCA) is possibly the most challenging cartilage procedure in the PFJ. As described for OAT, precise fitting of the harvested plug and creating a smooth surface is key to the success of the procedure. The same principle applies to OCA, but the process of donor graft matching is complicated by the anatomic complexity and variability of the PFJ. Proper assessment of preoperative imaging studies for PFJ allograft matching is yet to be determined [83, 84].

With stricter guidelines imposed by the FDA for allograft tissue acquisition and storage, the risk of disease transmission decreased and OCA has become increasingly popular as a primary or salvage procedure [24, 85]. Fresh osteochondral allografts are harvested within 24 h of donor’s death and are preserved at a temperature of 4 °C [86]. Current recommendations advise a storage time of up to 28 days, but ideal shelf life remains controversial [87, 88•]. This storage technique has shown adequate chondrocyte viability and is routinely used for OCA [86, 89].

**Fig. 4:**
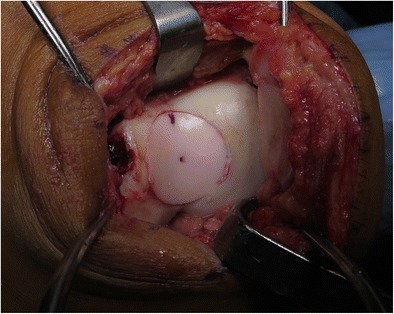
Intraoperative image of a flush seated osteochondral allograft to the trochlea. Marking of the 12 o’clock ensures correct insertion and thus perfect fitting of the osteochondral allograft plug

OCA is performed via an arthrotomy with a size and side-matched donor plug to precisely press fit into the prepared defect (Fig. 4). Despite the fact that allograft immunogenicity is related to graft volume, no differences were observed in survival rates for different graft sizes [90••]. Nevertheless, reducing the thickness of the graft’s subchondral bone is thought to reduce the potential risk of immunoreaction [83]. Additionally, pulse lavage washing of the graft is performed in an attempt to decrease marrow contents [91, 92].

OCA is indicated as a primary procedure in large full-thickness cartilage lesions, typically greater than 2 cm^2^, with abnormal subchondral bone [83], but increasingly also for the primary treatment of pure chondral lesions. Besides the prospect of addressing uncontained defects, OCA has the advantage of being a single-stage procedure while avoiding potential donor site morbidity compared with OAT. Moreover, it is a potential option for the treatment of bipolar lesions, but with inferior results when compared to outcomes in single lesions, similar to other cartilage repair options such as ACI [43•]. It can also serve as a salvage procedure in patients after failed cartilage repair and in patients with post-traumatic osteochondral defects after knee fractures that are too young for arthroplasty [86, 89, 93, 94]. Relative contraindications for OCA include smoking and obesity as they have shown to negatively affect clinical outcomes [87, 95•]. Extensor mechanism malalignment, ligamentous instability, or other intra-articular pathologies should be simultaneously addressed if needed to achieve improved outcomes.

There is a paucity in the current literature of randomized controlled trials for OCA, and only few studies have reported outcomes for patellofemoral OCA, albeit with good results [96]. Cameron et al. [42••] evaluated 29 knees with trochlear OCA with a mean follow-up of 7 years. Graft survivorship was 100% at 5 years and 91.7% at 10 years with improvement of all functional outcomes and an overall satisfaction rate of 89%. Gracitelli et al. [41••] studied 28 knees with OCA for isolated patellar cartilage lesions with 9.7 years of mean follow-up time. Patellar allograft survivorship was 78.1% at 5 and 10 years, decreasing to 55.8% at 15 years with an overall satisfaction rate of 89%. The obtained results are in conformity with outcomes reported by previous studies [97, 98].

OCA failure rate has been shown to be higher in the PFJ than in the TF. In a systematic review, Assenmacher et al. [99•] reported failure rates of 50 and 24% for OCA after a mean follow-up of 12.3 years in the PFJ and TF, respectively. Also, the reoperation rate was higher in the PFJ group when compared to the TF group (83 vs. 34%).

**Fig. 5:**
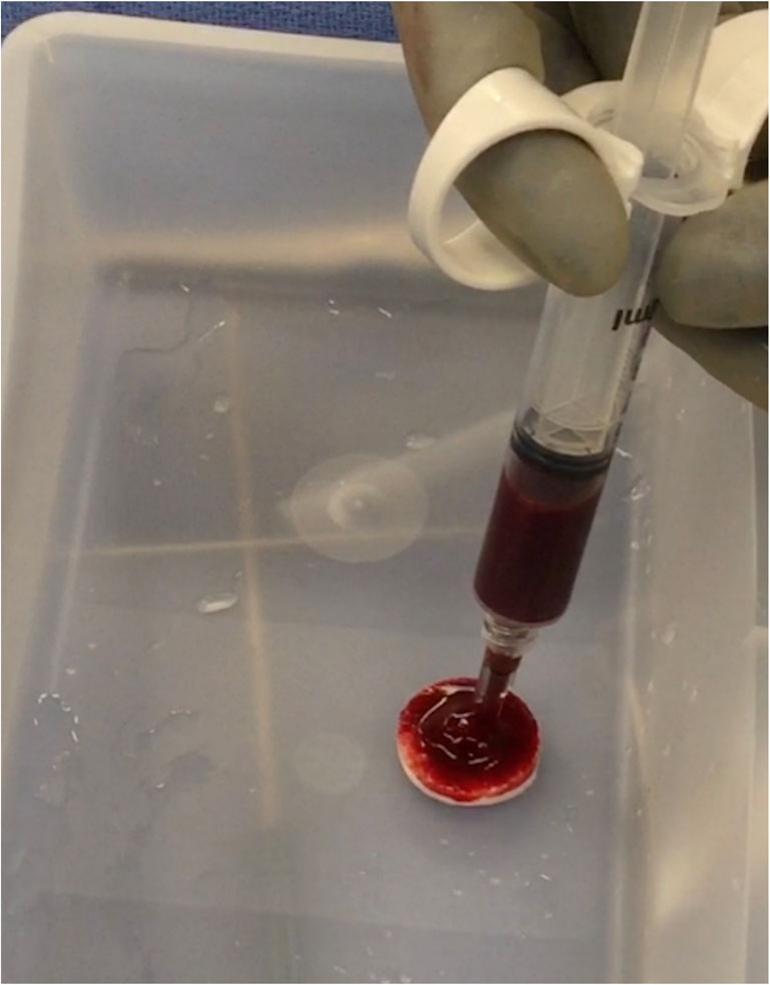
BMAC augmentation of an osteochondral allograft. Bone marrow is extracted from the lateral femoral condyle or tibial plateau

In terms of indication for OCA, the senior author distinguishes between chondral lesions affecting the patellar or trochlear articular surface. In the patella, OCA is typically performed after previously failed repair of large chondral lesions over 2 cm^2^ to provide pain relief and delay arthroplasty in young patients. In the trochlea, OCA can either be a primary treatment option for osteochondral lesions over 2 cm^2^ or serve as a salvage procedure after failed cartilage repair. Additionally, BMAC augmentation is frequently performed to enhance graft integration [46•, 95•] (Fig. 5).

### Particulated Cartilage Procedures

This relatively new treatment option includes particulated articular cartilage using autograft or juvenile allografts (DeNovo, Zimmer, Warsaw, IN) from donors aged 0–13 years [100•]. Analogous to OAT, articular cartilage is harvested from non-weight-bearing areas of the ipsilateral knee for the autograft procedure. Once the cartilage is mechanically minced, it is re-implanted into the chondral lesion and sealed with fibrin glue. A preclinical study demonstrated that particulated autograft loaded on composite scaffold produced hyaline-like cartilage [101]; one clinical study showed encouraging results for addressing lesions on the femoral condyle and trochlea [102].

**Fig. 6:**
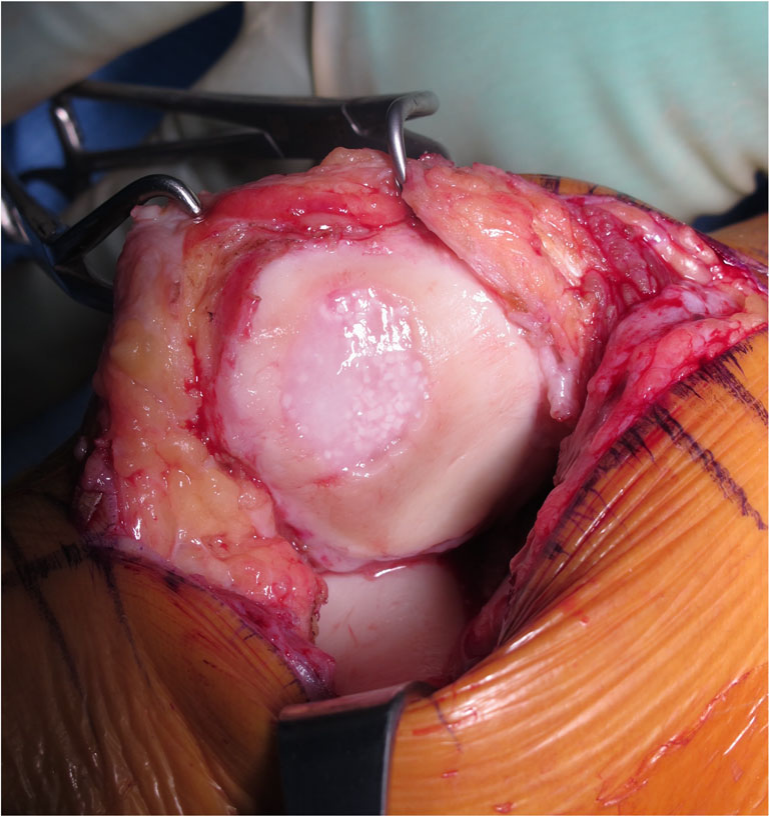
Intraoperative image of particulated juvenile osteochondral allograft to a chondral lesion on the patella

Particulated juvenile allograft is mechanically minced into 1–2 mm pieces and stored in vials containing enough material to cover lesions up to 2.5 cm^2^. Similar to autologous particulated cartilage, the chondral lesion is filled with particulated allograft up to 1 mm below the surrounding shoulders of healthy cartilage and finally secured with fibrin glue (Fig. 6). Indications include focal chondral lesions between 1 and 6 cm^2^ and ICRS grade 3 or higher in patients preferably younger than 55 years and a BMI less than 35 kg/m^2^ [100•].

There is a limited body of literature reporting clinical outcomes for particulated juvenile allograft transplantation. However, several authors demonstrated favorable results in patients with focal chondral lesions on the patella and trochlea [44•, 45•, 103], with good to excellent defect fill in short and mid-term results [45•, 104•]. Particulated juvenile articular cartilage has also been found to produce a mixture of hyaline and fibrocartilage with a dominance of type II collagen after 2 years follow-up [45•] (Table 2).

**Table 2:** Studies of auto- and allograft procedures for the treatment of chondral lesions in the PFJ

Study	LE	Cartilage repair technique	No. of patients	Mean patient age in years (range)	Mean follow-up (range)	Defect size, mean in cm^2^	Outcomes
Baltzer et al. [39•]	IV	OAT	112	48.01	26.2 months	2.55	Improvement in pain and quality of life. Retropatellar defects were associated with poor WOMAC (*p* = 0.03)
Astur et al. [40••]	IV	OAT	33	37.6 (16–59)	30.2 months (24–54)	1–2.5	Significant improvement in Lysholm, Fulkerson, and Kujala scores. 83% of plugs had complete osseous integration at 6 months, and 100% at 12 months
Gracitelli et al. [41••]	IV	OCA	27	33.7 (14–64)	9.7 years (1.8–30.1)	10.1	Survivorship was 78.1% at 5 and 10 years and 55.8% at 15 years. 89% of patients were extremely satisfied or satisfied
Cameron et al. [42••]	IV	OCA	28	30.2 (12–47)	7 years (2.1–19.9)	6.1	Graft survivorship was 100% at 5 years and 91.7% at 10 years. 89% of patients were extremely satisfied or satisfied
Meric et al. [43•]	IV	Bipolar OCA	46	40 (15–66)	7 years (2–19.7)	Median, 24.6 (failures) vs. 15.7 (non-failures)	Survivorship was 64.1% at 5 years. Patients with surviving allografts showed significant clinical improvement
Buckwalter et al. [44•]	IV	Particulated juvenile allograft	13	22.5 (14–34)	8.2 months (0.67–32.7)	2.05	Improvement in all KOOS subscores without any complications
Farr et al. [45•]	IV	Particulated juvenile allograft	25	37 (18–56)	2 years	2.7	Gradual improvement of all postoperative scores. Mean lesion fill increased from 43.5% at month 3 to 109.7% at 24 months

### Postoperative Rehabilitation

Postoperative protocols differ between institutions and remain a topic of great discussion. Although a standard postoperative rehabilitation protocol for PFJ cartilage repair is presented, each case should be carefully evaluated and the protocol adjusted as needed.

Generally, we recommend cryotherapy, elevation, and a brace for pain control during the immediate postoperative period. After 2 or 3 days, weight-bearing as tolerated is allowed in a locked brace, and continuous progressive passive motion is started. Range of motion (ROM) is encouraged to prevent arthrofibrosis with no restrictions. Therefore, with assist of continuous passive motion (CPM), we advance ROM gain to 90° of flexion as fast as patients tolerate it, at least 5° a day. In case of concomitant TTO, partial weight-bearing is recommended for 4–6 weeks. Return to sports is not advisable until 6 to 12 months after surgery based on senior author’s experience. There are no differences on the protocol for patellar or trochlear lesions.

## Conclusion

**Fig. 7:**
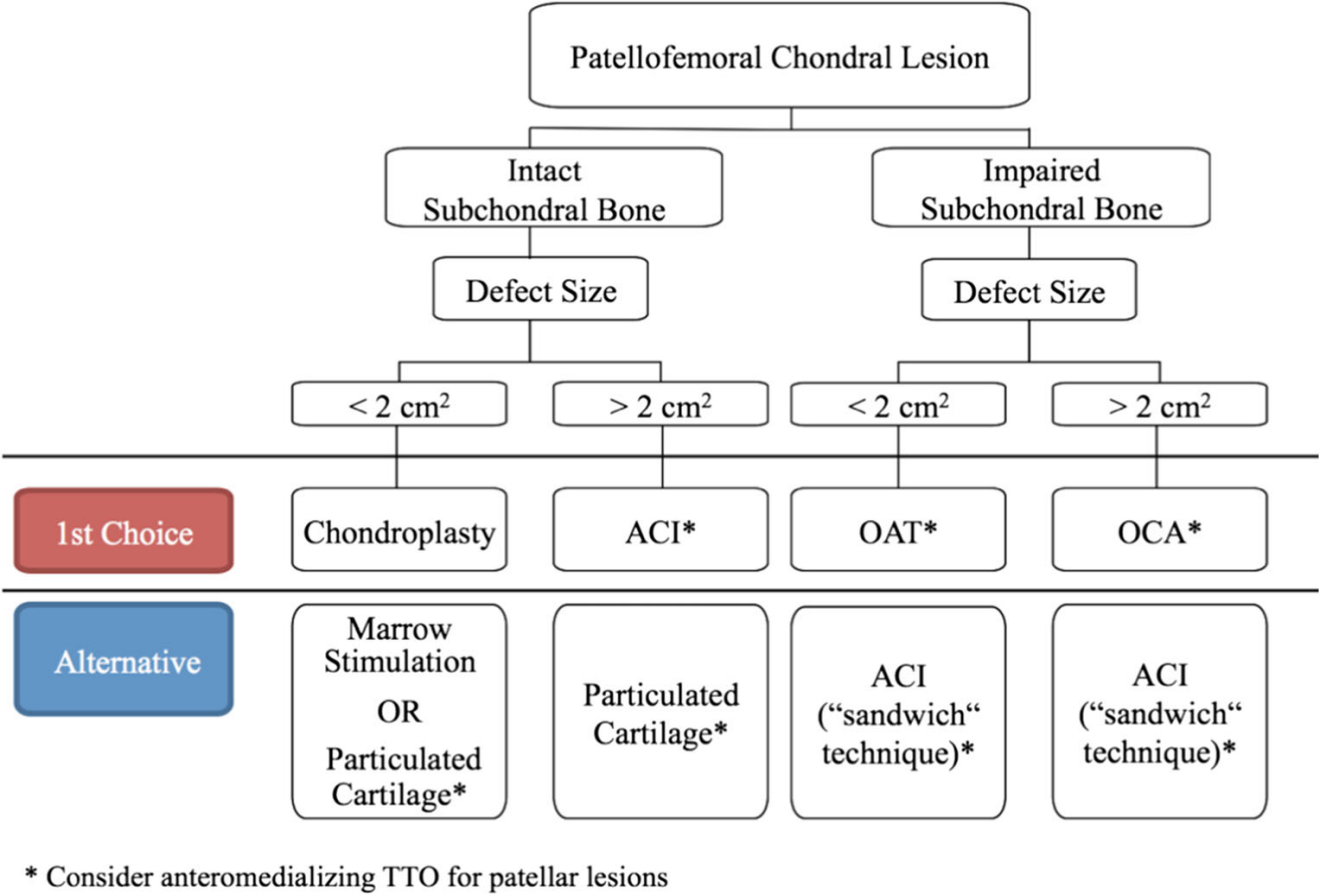
Treatment algorithm for patellofemoral chondral lesions

Cartilage repair in the PFJ has demonstrated increasingly good outcomes in patients with patellofemoral cartilage defects after conservative treatment has failed. The algorithm presented in Fig. 7

intends to serve as a simplified guide for the surgical management of chondral lesions in the PFJ. It is of utmost importance to discuss with the patient current functional limitations in sports and activities of daily living, to elucidate the patient’s goals and expectations, and to go over the rehabilitation and recovery time. Unrealistic expectations are common and will lead to disappointment. Careful evaluation of the knee and lower extremity through physical examination and imaging studies is crucial. This will allow planning a comprehensive treatment approach for the cartilage repair procedure, as well as any additional pathology that needs to be addressed in a staged or concomitant fashion.

The anatomic complexity and variability of the PFJ is a great challenge to all cartilage procedures. The increased attention to, and correction of, pathologic co-morbidities such as patellar instability and maltracking has led to a substantial improvement in results. While early outcomes of cartilage repair in the PFJ were disappointing, current comprehensive treatment approaches demonstrate outcomes that are comparable or only slightly inferior to cartilage repair in the TF.
